# ChatGPT/GPT-4: enabling a new era of surgical oncology

**DOI:** 10.1097/JS9.0000000000000451

**Published:** 2023-05-15

**Authors:** Kunming Cheng, Haiyang Wu, Cheng Li

**Affiliations:** a Department of Intensive Care Unit, The Second Affiliated Hospital of Zhengzhou University, Zhengzhou, Henan; b Department of Graduate School; c Clinical College of Neurology, Neurosurgery and Neurorehabilitation, Tianjin Medical University, Tianjin; d Department of Orthopaedic Surgery, Beijing Jishuitan Hospital, Fourth Clinical College of Peking University, Beijing, People’s Republic of China; e Duke Molecular Physiology Institute, Duke University School of Medicine, Durham, North Carolina, USA

*Dear Editor,*

Surgical oncology refers to the combination of basic knowledge and technology of modern surgery, as well as current tumor management principles, to carry out reasonable surgical resection of various tumor tissues. It is the main, if not the only, modality of cure for most solid cancers and an integral part of multimodality management of early and advanced-stage cancers. According to global statistics, out of the 15.2 million individuals newly diagnosed with cancer each year, more than 80% necessitate at least one surgical intervention throughout the course of their disease^1^. Benefiting from the development of surgical innovation and novel therapeutic approaches, applications of artificial intelligence (AI), and improvements in imaging techniques, surgical oncology has made remarkable progress in the past few decades.

Take AI as an example, machine learning algorithms and artificial neural networks are now renowned and widely used in image diagnosis and the construction of various clinical prediction models in surgical oncology practice. Of note, whether you are working in the field of AI or not, ChatGPT, a natural language processing chatbot assistant released by OpenAI in November 2022, must have captured your attention. As the fastest-growing consumer program in history, ChatGPT’s monthly active users have exceeded 100 million. More excitingly, one month ago, the release of the latest version, GPT-4, caused a sensation again. Compared with GPT-3.5, GPT-4 added many novel functions, such as processing more words and accepting image input^2^. Heralded as a disruptive technology, in the past half year, there have been more than 300 studies discussing the potential applications of ChatGPT/GPT-4 in multiple fields^3–4^. For example, Ayers *et al.* ^5^ evaluated the ability of ChatGPT to provide quality and empathetic responses to patient questions. They have found that the proportion of responses rated as good or very good quality was higher for ChatGPT than physicians, and chatbot responses were also rated significantly more empathetic than physician responses. However, to our knowledge, there is still a lack of reports to summarize the use of ChatGPT/GPT-4 in surgical oncology. In view of this, the present study discusses the potential application of ChatGPT/GPT-4 in this field from several perspectives.

Surgical oncology clinical trials, especially randomized control trials, are important means of evaluating operative outcomes. Unfortunately, clinical trials driven by surgeons have been traditionally poor and are still mainly made up of retrospective studies. The emergence of ChatGPT/GPT-4 may change this status quo in the following ways: The first is clinical trial design. AI technologies have unrivaled potential to collect, organize, and analyze the ever-increasing amounts of data generated by clinical trials, including failure data, to extract meaningful patterns of information to aid in design. ChatGPT/GPT-4 could assist oncological surgeons in evaluating the plausibility of clinical trials, including random grouping and sample size calculation, and also provide technical support on feasibility and innovation analysis, as well as statistical analysis. The second is case management and data analysis. After ChatGPT/GPT-4 access to case management systems, it could help oncological surgeons record and extract information such as patient history, examination results, treatment plans, and surgical procedures. In the meantime, in-depth analysis of clinical trial data, ChatGPT/GPT-4 could help researchers spot underlying patterns and trends in the surgical success rate and complication rate between different surgical approaches. In addition, as for other facets such as ethical review, patient education, and risk prediction models, ChatGPT/GPT-4 could also play an important role.

In recent decades, the clinical evaluation and management of cancer patients have undergone profound changes. With the deepening of research on the genetic and molecular mechanisms of malignant tumors, drugs targeting specific structures, functional regions, enzymes, and signal transduction pathways of tumor cells are increasingly being used clinically. In addition to conventional surgery, chemotherapy, and radiotherapy, targeted therapy and immunotherapy have emerged as important treatment modalities with encouraging results. Now more than ever, oncology surgeons need to understand the mechanisms and potential consequences of these novel therapies. As an encyclopedia, with self-renewal ability, ChatGPT/GPT-4 is able to quickly screen and summarize information from research literature and treatment guidelines, helping oncology surgeons understand the latest developments in these treatments.

During the preoperative preparation stage, including diagnostic imaging, multidisciplinary team (MDT) support, and surgical planning sessions, ChatGPT/GPT-4 could play a valuable role. For example, imaging and histological examinations are crucial for the diagnosis of tumors, affecting initial treatment and risk stratification. Previous findings showed that AI-based algorithms and tools exhibited high performance in the diagnosis and intelligent classification of multiple tumors. The combination of medical imaging and AI tools, such as ChatGPT/GPT-4, is considered the most promising field. Take tumor area recognition as an example; in the traditional approach, tumor areas were manually delineated based on the radiologist’s experience, which is easily influenced by subjectivity, leading to inconsistent views between the radiologist and oncologic surgeon. While an automated delineation of tumor borders by an AI algorithm provided reproducible results, which not only reduced the burden on occupational physicians but also gave an important reference for preoperative planning. Therefore, in the future, ChatGPT/GPT-4 could be used to comprehensively analyze clinical data, histological data, imaging data, and even genetic data of tumor patients and further optimize the tumor recognition and classification methods.

In addition, the MDT treatment strategy is an important part of cancer therapy. The UK clinical practise guidelines in oncology state that all patients diagnosed with tumors must undergo relevant MDT consultations before receiving treatment. However, in practical work, there are still some difficulties in interdisciplinary collaboration, such as inconsistent diagnosis and treatment plans. ChatGPT/GPT-4 could master the knowledge of various medical disciplines if they received adequate medical information and training. Thus, it can be inferred that ChatGPT/GPT-4 may become an essential member and even serve as an alternative to well-trained medical specialists to provide accurate, rapid surgical recommendations.

As for intraoperative processes, traditional tumor resection surgery is performed based on imaging results and the surgeon’s preference. Oncologic surgeons usually judge the tumor boundary based on personal experience. Excessive resection will increase the risk of normal tissue damage and cause unnecessary injury. Insufficient resection will not completely remove the tumor mass as thoroughly as possible, resulting in residual neoplastic tissues. Therefore, preoperative simulation has become an essential step in preoperative planning. Firstly, ChatGPT/GPT-4 enables simulation of the tumor resection process, which assists young surgeons in conducting virtual surgery simulations and improving their surgical skills. Secondly, AI tools, such as ChatGPT/GPT-4 based anatomical landmark navigation, are expected to bring value to future surgeries by identifying the correct anatomical plane and avoiding unintended organ damage. Thirdly, the robot-assisted surgical system has been extensively employed in surgical oncology. However, surgical robots still require human manipulation. In the future, ChatGPT/GPT-4 could become the brain of the surgical robot instead of surgeons. Surgical robots can be considered an advanced version of ChatGPT/GPT-4 in surgical oncology.

All in all, ChatGPT/GPT-4 could involve the entire process of surgical oncology, and a new era in this area has been coming. Although it is undeniable that ChatGPT/GPT-4 is a potent tool to enhance the productivity and effectiveness of oncological surgeons, being mindful of its potential negative consequences is essential. Recently, the news keeps coming out that many institutions and counties are planning or have already announced the prohibition of the use of ChatGPT/GPT-4 in academic and social activities due to safety and ethical concerns. In our view, this strategy is myopic. The development of AI has become an inexorable trend. Therefore, instead of a complete prohibition, it is better to establish guidelines for responsible and effective use.

## Ethical approval

This study does not include any individual-level data and thus does not require any ethical approval.

## Sources of funding

This study is supported by China Postdoctoral Science Foundation (2022M720385) and Beijing JST Research Funding (YGQ-202313).

## Author contribution

K.C.: methodology, data curation, formal analysis, resources, investigation, writing – original draft, and writing – review and editing; H.W.: conceptualization, methodology, data curation, formal analysis, resources, investigation, writing – original draft, and writing – review and editing; C.L.: conceptualization, formal analysis, resources, investigation, and writing – review and editing.

## Conflicts of interest disclosure

The authors declare no conflicts of interest.

## Research registration unique identifying number (UIN)

1. Name of the registry: not applicable.
2. Unique identifying number or registration ID: not applicable.
3. Hyperlink to your specific registration (must be publicly accessible and will be checked): not applicable.

## Guarantor

Haiyang Wu and Cheng Li.

## Data availability statement

The data underlying this article will be shared by the corresponding author upon reasonable request.
